# Advances in the Treatment of Androgenetic Alopecia: A Review of Mechanisms and the Clinical Efficacy of Combination Drug Therapy

**DOI:** 10.1111/jocd.70926

**Published:** 2026-06-11

**Authors:** Zhuhao Lai, Yi Zhou, Rui Zhou

**Affiliations:** ^1^ Department of Plastic Surgery The Third Affiliated Hospital of Zhejiang Chinese Medical University, Zhejiang Chinese Medical University Hangzhou China; ^2^ Center for Plastic & Reconstructive Surgery, Department of Plastic & Reconstructive Surgery Zhejiang Provincial People's Hospital (Affiliated People's Hospital), Hangzhou Medical College Hangzhou China

**Keywords:** androgenetic alopecia, clinical efficacy, combination drug therapy, finasteride, mechanisms, minoxidil, spironolactone

## Abstract

**Background:**

Androgenetic alopecia (AGA) is the most prevalent type of hair loss, exerting a significant negative impact on patients' quality of life. The limited efficacy, potential adverse effects, and poor patient adherence of single‐agent therapies highlight substantial unmet needs in clinical management. Recently, combination drug therapy has emerged as a promising strategy and garnered increasing clinical attention.

**Objective:**

The aim is to provide a scientific basis for evidence‐based clinical practice and to facilitate the implementation of combined therapeutic strategies.

**Methods:**

This review systematically elucidates the pharmacological mechanisms of commonly used agents and explores the efficacy and safety of various combination regimens.

**Results:**

AGA is a multifactorial disorder. Combination therapy can enhance hair growth more effectively through distinct mechanisms while reducing the side effects of individual medications by lowering their respective dosages. Furthermore, the formulation of personalized combination regimens enables patients with varying etiologies, genders, and AGA severity levels to achieve improved outcomes. This tailored approach enhances patient compliance, thereby optimizing therapeutic efficacy.

**Conclusion:**

Combination pharmacotherapy represents a significant advancement in the treatment of AGA, offering enhanced efficacy and the potential for individualized patient care.

## 1 Introduction

Androgenetic alopecia (AGA) is a chronic, progressive, non‐scarring hair loss disorder that affects a substantial proportion of both men and women worldwide, with prevalence rates increasing with age and reaching up to 80% of males and 40% of females over their lifetimes [1, 2]. The pathogenesis of AGA is multifactorial, involving a complex interplay between genetic predisposition and androgenic activity. Key mechanisms include the upregulation of 5‐α‐reductase and androgen receptors within hair follicles, leading to the miniaturization of terminal hairs and a shortened anagen phase [1, 3]. In addition to these primary factors, recent studies have highlighted the roles of low‐grade inflammation, oxidative stress, depletion of hair follicle stem cells, and associations with metabolic syndrome in the disease process [1, 4]. Besides, the psychosocial impact of AGA is considerable, often leading to diminished self‐esteem, social withdrawal, and reduced quality of life, particularly among younger patients and those actively seeking treatment [5, 6].

Current therapeutic strategies for AGA remain limited, with only two FDA‐approved medications: topical minoxidil and oral finasteride [1, 7]. Despite their widespread use, these monotherapies often yield suboptimal results, with many patients experiencing limited regrowth, plateauing efficacy, or adverse effects [1, 8]. The chronic nature of AGA necessitates long‐term adherence to therapy, which can be challenging due to these limitations and the need for continuous application or ingestion to maintain benefits [2]. Additionally, a subset of patients exhibits resistance or intolerance to standard treatments, further underscoring the need for alternative or adjunctive modalities [1, 7].

In response to the limitations of monotherapy, combination and multimodal treatment strategies have gained increasing attention. The rationale for combination therapy lies in the multifactorial pathogenesis of AGA, in which targeting multiple pathways may yield synergistic effects, enhance efficacy, minimize adverse effects, and delay the development of drug resistance [1, 9]. Emerging evidence supports the use of combined pharmacological regimens—such as finasteride with minoxidil, or the addition of anti‐androgens, platelet‐rich plasma (PRP), microneedling, and low‐level laser therapy (LLLT)—to achieve superior clinical outcomes compared with monotherapy [10, 11, 12]. Novel agents, including topical anti‐androgens, stem cell‐derived therapies, and natural compounds with anti‐inflammatory or antioxidant properties, are also under investigation as adjuncts or alternatives to conventional drugs [3, 13]. This evolving therapeutic landscape reflects a paradigm shift towards individualized, mechanism‐based, and patient‐centred management of AGA.

This article aims to provide a systematic overview of the mechanisms, clinical efficacy, and safety profiles of combined drug therapies for AGA. It will examine the molecular and clinical rationale for multi‐target interventions, summarize key findings from recent clinical trials, and discuss practical considerations and future directions for optimizing AGA management through combination approaches.

## 2 Main Text

### 2.1 Mechanisms of Drug Therapy and Their Limitations

#### 2.1.1 Finasteride

Finasteride is a selective inhibitor of type II 5‐α‐reductase, the enzyme responsible for converting testosterone into dihydrotestosterone (DHT). By reducing DHT levels, finasteride mitigates androgen‐mediated miniaturization of hair follicles, thereby slowing the progression of hair loss and promoting hair regrowth in affected individuals [14, 15]. However, finasteride's use is primarily limited to male patients, and the most notable limitation of oral finasteride is associated with sexual dysfunction, including decreased libido, erectile dysfunction, and ejaculatory disorders [14, 16, 17, 18]. A meta‐analysis incorporating 34 randomized controlled trials revealed that patients using 5α‐reductase inhibitors (including finasteride) had a 1.89‐fold higher risk of experiencing sexual adverse effects compared to the placebo group [19]. In addition, reports of mood changes, depression, and cognitive dysfunction have raised concerns about its broader safety profile [20, 21, 22]. Plasma concentrations following topical administration of finasteride are two orders of magnitude lower than those observed with oral dosing. This reduced level of systemic exposure implies a substantially lower risk of systemic adverse effects compared to the oral formulation [23, 24]. Thus, while finasteride remains a mainstay of pharmacological management of AGA in men, its use is tempered by patient selection, risk of adverse effects, and the need for ongoing monitoring and informed consent.

#### 2.1.2 Minoxidil

Topical minoxidil is a cornerstone therapy for AGA and is FDA‐approved for both male‐ and female‐pattern hair loss [25, 26]. Its mechanism of action is multifaceted. Primarily, minoxidil acts as a vasodilator by opening ATP‐sensitive potassium channels in vascular smooth muscle, thereby increasing blood flow around hair follicles and improving follicular nutrition [27, 28]. In addition, minoxidil may exert anti‐inflammatory effects, stimulate the Wnt/β‐catenin signaling pathway, and modulate androgen receptor activity. Recent studies have identified the role of CYP17A1 and CYP19A1 in hormonal and enzymatic pathways that further suppress androgenic effects on hair follicles [29]. For minoxidil to be effective, it must be converted to its active form, minoxidil sulfate, by the enzyme sulfotransferase (SULT1A1). Variability in SULT1A1 activity among individuals accounts for the heterogeneity in clinical response, and efforts to enhance this enzyme's activity have shown promise in improving treatment outcomes [30, 31].

The local adverse effects of minoxidil include irritant and allergic contact dermatitis, pruritus, and, less commonly, hypertrichosis of the face or ears [26, 32, 33]. There is limited safety data on the long‐term use of oral minoxidil, but it has been suggested that long‐term use may lead to hypotension, tachycardia, and systemic hirsutism [34]. Patient adherence to topical minoxidil remains a considerable challenge owing to the need for daily, long‐term application and cosmetic concerns such as greasiness or residue [35, 36, 37]. Discontinuation of minoxidil typically results in the resumption of hair loss within months [25]. Furthermore, a subset of patients experiences minimal or no response, often attributed to insufficient conversion to minoxidil sulfate or inter‐individual variation in follicular sensitivity [30, 31]. In summary, while topical minoxidil is an effective and generally well‐tolerated option for many, its efficacy is constrained by the requirement for continuous use, the risk of relapse after cessation, and variable patient response.

#### 2.1.3 Spironolactone and Other Antiandrogenic Agents

Spironolactone is an oral antiandrogenic medication that has gained widespread use, particularly in female patients. Its mechanism of action involves antagonism of androgen receptors, thereby reducing the impact of circulating androgens on hair follicles and halting the progression of hair loss [38]. A meta‐analysis reported that the overall incidence of adverse events associated with spironolactone treatment for hair loss was 3.69%. The most commonly reported adverse effects included scalp pruritus or increased dandruff, as well as menstrual disorders, facial hypertrichosis, and drug discontinuation [39]. However, spironolactone's use in men is markedly limited owing to the risk of feminizing adverse effects, including gynaecomastia, decreased libido, and erectile dysfunction [38, 40]. Importantly, recent studies have indicated that spironolactone does not increase the risk of breast cancer recurrence, supporting its safety in high‐risk female populations [41, 42]. Other antiandrogenic agents, such as bicalutamide, flutamide, and cyproterone acetate, have also been investigated for AGA, but concerns regarding safety profiles and long‐term risks persist, particularly in populations with a history of hormone‐sensitive malignancies [41]. Overall, spironolactone remains a cornerstone of antiandrogenic management for female AGA, while long‐term monitoring and the development of novel topical antiandrogens continue to expand therapeutic options for both sexes [38, 43].

### 2.2 Theoretical Basis and Mechanistic Rationale for Combination Therapy

#### 2.2.1 Multi‐Target Synergistic Effects

The combination of finasteride and minoxidil for the treatment of AGA exemplifies a multi‐target synergistic therapeutic strategy that addresses both the underlying hormonal and follicular pathophysiology of hair loss. Finasteride suppresses the androgenic trigger, while minoxidil supports follicular regeneration and growth. A recent single‐blind study comparing topical finasteride 0.25% plus minoxidil 5% with minoxidil 5% alone in men with AGA demonstrated a significantly higher efficacy rate in the combination group (86.7% vs. 69.1%, *p* = 0.006), indicating that simultaneously targeting DHT production and follicular stimulation yields better hair regrowth outcomes [44]. Furthermore, nanostructured carriers have been shown to enhance the local bioavailability of agents, thereby improving therapeutic results while minimizing systemic exposure and adverse effects [45]. Additionally, microneedling has been shown to augment the efficacy of topical therapies in AGA [11]. The rationale for combining finasteride and minoxidil is therefore grounded in their distinct but complementary mechanisms—DHT suppression and follicular activation—which together address the multifactorial nature of AGA more effectively than monotherapy.

#### 2.2.2 Reducing Adverse Effects and the Risk of Monotherapy Resistance

Combination therapy for the treatment of AGA offers a promising approach to mitigating the adverse effects and resistance risks associated with monotherapy. By employing combination regimens, clinicians can use lower doses of each drug, thereby reducing the incidence and severity of adverse effects. Studies have shown that combining topical finasteride with minoxidil achieves similar efficacy in improving hair density and thickness as monotherapy, while minimizing systemic adverse effects such as reduced libido and erectile dysfunction [46]. In addition, the integration of microneedling or PRP with topical minoxidil has demonstrated enhanced hair‐regrowth outcomes with only mild and transient adverse effects, such as scalp erythema or pruritus [47, 48]. The combination of antiandrogens and growth factor‐based therapies could help maintain efficacy by targeting multiple pathogenic pathways [9, 49]. Overall, the evidence indicates that rational combination therapy not only enhances clinical effectiveness but also plays a crucial role in reducing adverse effects and slowing the progression of resistance, thereby improving patient compliance and long‐term treatment success in AGA.

#### 2.2.3 Individualized Treatment Strategies

The management of AGA is increasingly moving towards individualized treatment strategies, which take into account key patient‐specific factors such as sex, degree of hair loss, drug tolerability, and comorbidities to optimize outcomes and enhance adherence and satisfaction. Genetic background and ethnic differences further influence treatment response, particularly in female‐pattern hair loss, highlighting the need for genetic screening and personalized regimens [50]. The severity of AGA also guides treatment selection and intensity; patients with more advanced hair loss may require combination therapies, such as minoxidil with finasteride or adjunctive microneedling, to achieve meaningful improvements [11, 44]. Furthermore, patient tolerability and risk of adverse effects must be carefully considered—topical formulations or lower concentrations are preferable for those with heightened sensitivity [51]. Innovations in drug delivery also enable customization of active ingredients and dosing [52, 53]. Psychosocial factors also influence adherence and satisfaction, suggesting that individualized care should extend beyond pharmacological choices to encompass patient education and support [54]. Additionally, scalp‐microbiome profiling and machine learning offer promising avenues for early diagnosis and truly personalized interventions [55]. Collectively, these advances underscore the need for clinicians to adopt a holistic, patient‐centered approach, integrating clinical, genetic, psychosocial, and technological factors to design individualized combination therapies that maximize efficacy, minimize adverse effects, and improve overall patient satisfaction in the management of AGA.

### 2.3 Advances in Clinical Research on Combination Therapy

#### 2.3.1 Clinical Efficacy of Finasteride–Minoxidil Combination Therapy

Multiple randomized controlled trials and meta‐analyses have demonstrated that the combination of finasteride and minoxidil yields superior clinical outcomes in AGA compared with monotherapy (Table 1). A systematic review and meta‐analysis reported that patients receiving combined finasteride and topical minoxidil therapy achieved significantly higher global photographic evaluation scores, a greater proportion of patients with marked improvement, and fewer cases of deterioration or no change than those treated with either agent alone. Importantly, the combination group did not experience an increased risk of adverse events, indicating that the enhanced efficacy was not accompanied by compromised safety [10]. Similarly, Li et al. conducted a meta‐analysis incorporating seven randomized controlled trials (RCTs) and reported that the combined topical minoxidil‐finasteride solution was significantly superior to minoxidil monotherapy in improving hair density (mean difference [MD] = 9.22, *p* = 0.04), hair diameter (MD = 2.26, *p* = 0.005), and overall photographic assessment (MD = 0.79, *p* < 0.00001) [65]. In addition, a pilot randomized open‐label study found that the group treated with topical minoxidil 5% and finasteride 0.25% demonstrated significantly greater improvements in both total and terminal hair density at 24 weeks compared with monotherapy groups (*p* < 0.02 and *p* < 0.001) [56]. A large retrospective evaluation of more than 500 men treated with a combined oral low‐dose minoxidil and finasteride regimen over 12 months reported that over 92% achieved stable or improved outcomes and nearly 60% showed marked improvement, with particularly robust responses observed in patients with more severe baseline hair loss [57]. Notably, combination therapy has been associated with significant improvements as early as 3 months after initiation [44]. Collectively, the evidence supports the use of combined finasteride and minoxidil therapy as an effective strategy in the management of AGA [10, 44, 56, 57, 58].

**TABLE 1:** Comparative efficacy of monotherapies and combination therapies for androgenetic alopecia.

Author(s), year	Study design	F/M	Group(s)	Follow‐up	Assessment	Outcomes	Side effects
Finasteride‐minoxidil							
Nazia Asad et al. (2024) [44]	RCT, single‐blind	0/164	Topical Finasteride 0.25% With Minoxidil 5% Topical Minoxidil 5% Alone	12 weeks	Hair regrowth	86.7% vs. 69.1%; *p* = 0.006	No
Bharadwaj et al. (2023) [56]	Pilot randomized open‐label study	0/60	Topical 5% minoxidil and 0.25% finasteride combination (MNF) 5% minoxidil (MNX) 0.25% finasteride (FNS)	24 weeks	Hair count, physician assessment score (PAS), and patient satisfaction score (PSS).	MNF was comparatively superior (*p* = 0.028 in 12 week, and *p* < 0.02 in 24 week)	Scaling and itching were reported with MNF and MNX.
Hans Johnson et al. (2025) [57]	Retrospective study	0/502	Combined oral low‐dose minoxidil‐finasteride	12 months	Hair density	Statistically significant and clinically meaningful improvements (*p* < 0.001)	No
Lubis et al. (2025) [46]	RCT	0/40	Topical finasteride 0.1%‐minoxidil 5% (treatment) Topical minoxidil 5% (control)	12 weeks	Hair density, hair diameter, terminal hair rate, and vellus hair rate	No differences between groups	No difference
Rossi A et al. (2024) [58]	RCT	0/42	5% minoxidil and finasteride spray finasteride spray (F) 5% minoxidil (MNX)	6 months	Hair density	Only group A (MNX + F) showed improvement both at three (+56 density/cm^2^, *p* < 0.05) and six (+81 density/cm^2^, *p* < 0.001) months	No
Spironolactone‐minoxidil							
Liang X et al. (2022) [49]	RCT	120/0	minoxidil (MX) MX + spironolactone (SPT) MX + microneedling (MN)	24 weeks	Hair growth (hair density and diameter), scalp tissue structure	Hair density increased most in MX + MN group and increased least in MX group (*p* < 0.001 for MX + MN group vs. MX + SPT group; *p* = 0.009 for MX + SPT group vs. MX group).	The most adverse effects were reported in MX + SPT group (*n* = 45).
Desai et al. (2024) [59]	Retrospective study	21/0	Low‐dose oral minoxidil (LDOM) with spironolactone LDOM with finasteride/dutasteride	142.0 or 88.9 days	Hair width and density	LDOM with finasteride/dutasteride group showed a greater average increase (*p* > 0.05)	No difference
Sadeghzadeh et al. (2024) [60]	RCT	60/0	Minoxidil 2% and spironolactone 100 mg/day Minoxidil 2% and finasteride 5 mg/day	4 months	Hair loss, physician satisfaction	Spironolactone produced better results in hair density than finasteride (*p* < 0.05)	Two cases in spironolactone group report menstrual irregularities
Others							
Chang et al. (2025) [61]	RCT	0/45	A: 5% Minoxidil B: 5% Minoxidil and Finasteride C: microneedling, 5% Minoxidil, and Finasteride	6 months	Hair density and hair shaft diameter	Group B and group C was superior to group A (*p* < 0.05), group C was superior to group B (*p* > 0.05)	No difference
Muhammad et al. (2022) [62]	RCT	3/57	PRP + microneedling PRP alone	/	Hair count	Hair count in the microneedling group (24.53% ± 9.49%) was significantly higher than the increase in the PRP‐alone group (17.88% ± 10.15%) (*p* = 0.011)	/
Charoensuksira et al. (2024) [63]	Comparative prospective study	12/4	LED combined with LMNPs	6 months	Hair density and diameter	Significantly enhanced hair density and diameter (*p* < 0.05)	No
Wang et al. (2023) [64]	Retrospective, case‐series study	0/9	PRP and HA	6 months	Hair loss, hair count, treatment satisfaction	Substantial increases in hair density and high satisfaction rates	/

#### 2.3.2 Combined Use of Spironolactone and Minoxidil in Female AGA

The combination of spironolactone and minoxidil has emerged as a promising therapeutic strategy for female AGA. Evidence from a systematic review indicates that both oral and topical spironolactone are effective for alopecia recovery, and their efficacy is further improved when combined with minoxidil, regardless of the route of administration [38]. A randomized controlled trial in women with mild‐to‐moderate female‐pattern hair loss found that the group receiving 5% topical minoxidil plus oral spironolactone (80–100 mg/day) experienced significantly greater increases in hair density compared with the minoxidil‐only group (16.76 ± 11.75/cm^2^ vs. 9.95 ± 10.16/cm^2^, *p* = 0.009), and the combination with microneedling produced the most pronounced effect (30.33 ± 15.18, *p* < 0.001). Adverse effects in the minoxidil plus spironolactone group were generally mild and manageable [49]. Sadeghzadeh et al. reported spironolactone produced better results than finasteride in the treatment with female AGA [60]. However, Deesha et al. founded low‐dose oral minoxidil in combination with spironolactone has shown comparable efficacy and safety profiles when assessed against combinations with other antiandrogens, such as finasteride or dutasteride, reinforcing the role of this dual therapy as an effective and safe approach for women [59]. These findings collectively support the use of spironolactone and minoxidil in combination for female AGA (Table 1). However, ongoing research and long‐term studies are warranted to further optimize dosing strategies and confirm the durability of clinical benefits.

#### 2.3.3 Other Emerging Combination Regimens

Recent research has expanded the investigation of combination regimens for AGA beyond traditional pharmacological approaches, incorporating anti‐inflammatory agents, laser therapies, regenerative medicine, and novel drug‐delivery technologies to enhance therapeutic outcomes (Table 1). LLLT and microneedling are also gaining traction as adjuncts to standard treatments [61]. The use of microneedling in combination with topical minoxidil has shown a significant synergistic effect, producing greater increases in hair count compared with monotherapy, due to enhanced drug penetration and activation of growth factors through the wound‐healing cascade [66, 67]. Similarly, innovative devices such as light‐emitting diode (LED) helmets combined with light‐guiding microneedle patches (LMNPs) have demonstrated significant improvements in hair density and diameter, with high patient satisfaction and minimal adverse events, supporting their potential as noninvasive and effective combination therapies [63].

Furthermore, regenerative medicine strategies are showing promise. The combination of PRP with basic fibroblast growth factor and minoxidil has been found to be more effective than either treatment alone [48]. Likewise, pairing PRP with non‐cross‐linked hyaluronic acid (HA) compounds has resulted in substantial increases in hair density (54.51% at the 1‐month and 77.25% at the 3‐month follow‐up) and high satisfaction rates in women with AGA [64]. Nanotechnology‐based approaches are also emerging, with microneedle patches loaded with ROS‐scavenging nanozymes or pro‐angiogenic factors offering targeted delivery to hair follicles, alleviating oxidative stress, restoring the follicular microenvironment, and promoting perifollicular angiogenesis, thereby enhancing hair regeneration [68, 69]. In addition, microneedle‐mediated delivery systems that integrate photothermal therapy with herbal agents further potentiate follicular targeting and tissue remodeling, providing multifunctional, synergistic strategies for the management of AGA [62, 70]. Collectively, these novel combination regimens illustrate a trend towards multimodal, mechanism‐driven therapies that harness the synergistic effects of anti‐inflammatory, regenerative, and physical modalities to achieve superior clinical outcomes in AGA.

### 2.4 Management Considerations for Combination Therapy

Combination therapy for AGA presents unique challenges and opportunities with respect to patient adherence and medication management. Studies have shown that complex or burdensome medication schedules, frequent dosing, and adverse effects can reduce adherence rates in patients with AGA [71, 72]. Simplifying the medication regimen can reduce the frequency of administration, minimize adverse effects, and thereby improve adherence [73, 74, 75]. Furthermore, patient education plays a pivotal role; clear communication regarding the necessity of sustained treatment, potential adverse effects, and realistic expectations for hair regrowth is essential to foster long‐term commitment [71]. Ultimately, optimizing combination therapy for AGA requires a multifaceted approach: simplifying regimens through innovative pharmaceutical formulations, proactive management of adverse effects, and robust patient education and support programs, all of which have been shown to enhance compliance and improve clinical outcomes [9, 76].

## 3 Limitations of the Current Evidence

(1) the paucity of studies with follow‐up beyond 12–24 months, (2) the methodological heterogeneity across trials which complicates direct comparison, (3) the variability in primary endpoints (e.g., hair count vs. global photography), and (4) the potential for publication bias, particularly within the cosmetic dermatology field, and its implications for interpreting the reported efficacy.

## 4 Conclusion

Combination pharmacotherapy marks a significant advancement in the management of androgenetic alopecia (AGA), providing superior efficacy compared to monotherapy through synergistic mechanisms. Studies consistently demonstrate that regimens such as finasteride with minoxidil or spironolactone with minoxidil enhance hair regrowth, patient satisfaction, and long‐term disease control. These combinations are particularly beneficial for patients with advanced disease, rapid progression, or inadequate response to single‐agent treatment.

This multi‐target approach allows for individualized strategies based on sex, age, comorbidities, and risk tolerance, aligning with precision medicine principles. However, polypharmacy necessitates careful safety management and adherence support due to potential drug interactions and cumulative adverse effects. Shared decision‐making and patient education are essential to ensure understanding of treatment rationale and early adverse event recognition.

While current evidence supports the clinical value of combination therapy, larger multicenter long‐term trials are needed to validate efficacy and safety across diverse populations. Emerging therapies—such as selective androgen receptor modulators and microRNA‐based treatments—may further expand combination strategies.

## Funding

This work was supported by China Postdoctoral Science Foundation (2024M752888) and Natural Science Foundation of Zhejiang Province (LQN25H150001).

## Ethics Statement

The authors have nothing to report.

## Conflicts of Interest

The authors declare no conflicts of interest.

## Data Availability

Data sharing not applicable to this article as no datasets were generated or analysed during the current study.
